# Whole-Body Magnetic Resonance Imaging (MRI) for Staging Melanoma Patients in Direct Comparison to Computed Tomography (CT): Results from a Prospective Positron Emission Tomography (PET)/CT and PET/MRI Study

**DOI:** 10.3390/diagnostics13111963

**Published:** 2023-06-05

**Authors:** Christian Philipp Reinert, Cecilia Liang, Matthias Weissinger, Jonas Vogel, Andrea Forschner, Konstantin Nikolaou, Christian la Fougère, Ferdinand Seith

**Affiliations:** 1 Department of Radiology, Diagnostic and Interventional Radiology, University Hospital Tübingen, Hoppe-Seyler-Str. 3, 72076 Tübingen, Germany; 2 Department of Nuclear Medicine and Clinical Molecular Imaging, University Hospital Tübingen, Hoppe-Seyler-Str. 3, 72076 Tübingen, Germany; 3 Department of Dermatology, University Hospital Tübingen, Liebermeisterstrasse 25, 72076 Tübingen, Germany; 4 Cluster of Excellence iFIT (EXC 2180) “Image Guided and Functionally Instructed Tumor Therapies”, University of Tübingen, 72076 Tübingen, Germany; 5 German Cancer Consortium (DKTK), Partner Site Tübingen, 72076 Tübingen, Germany

**Keywords:** melanoma, computed tomography, magnetic resonance imaging, positron emission tomography, staging

## Abstract

Purpose: The consideration of radiation exposure is becoming more important in metastatic melanoma due to improved prognoses. The aim of this prospective study was to investigate the diagnostic performance of whole-body (WB) magnetic resonance imaging (MRI) in comparison to computed tomography (CT) with ^18^F-FDG positron emission tomography (PET)/CT and ^18^F-PET/MRI together with a follow-up as the reference standard. Methods: Between April 2014 and April 2018, a total of 57 patients (25 females, mean age of 64 ± 12 years) underwent WB-PET/CT and WB-PET/MRI on the same day. The CT and MRI scans were independently evaluated by two radiologists who were blinded to the patients’ information. The reference standard was evaluated by two nuclear medicine specialists. The findings were categorized into different regions: lymph nodes/soft tissue (I), lungs (II), abdomen/pelvis (III), and bone (IV). A comparative analysis was conducted for all the documented findings. Inter-reader reliability was assessed using Bland–Altman procedures, and McNemar’s test was utilized to determine the differences between the readers and the methods. Results: Out of the 57 patients, 50 were diagnosed with metastases in two or more regions, with the majority being found in region I. The accuracies of CT and MRI did not show significant differences, except in region II where CT detected more metastases compared to MRI (0.90 vs. 0.68, *p* = 0.008). On the other hand, MRI had a higher detection rate in region IV compared to CT (0.89 vs. 0.61, *p* > 0.05). The level of agreement between the readers varied depending on the number of metastases and the specific region, with the highest agreement observed in region III and the lowest observed in region I. Conclusions: In patients with advanced melanoma, WB-MRI has the potential to serve as an alternative to CT with comparable diagnostic accuracy and confidence across most regions. The observed limited sensitivity for the detection of pulmonary lesions might be improved through dedicated lung imaging sequences.

## 1. Introduction

Over the last decades, the global incidence of melanoma has been steadily increasing, particularly among fair-skinned Caucasian populations [1]. While the occurrence of melanoma rises with age, peaking in the seventh and eighth decades of life, it is also a common cancer diagnosed in adolescents and young adults [2,3]. The early detection of regional and distant metastases during primary staging and follow-up examinations is crucial for one’s prognosis; however, there are still variations in the recommendations for the optimal choice of imaging method across different countries [4]. According to the 2019 European consensus-based interdisciplinary guideline for melanoma, whole-body examinations using CT or PET/CT in combination with brain MRI are recommended for stage IIC melanoma and above [5]. Follow-up examinations for patients with stage IIC to IIIC melanoma are suggested to be scheduled every 6 months in the first 3 years, every 3–6 months for stage IIID, and every 3 months for stage IV. Recent advancements in immunotherapies have led to improved overall survival, even in patients with advanced melanoma, with survival exceeding 60 months [6]. Considering the longer life expectancy of younger patients [7], it is essential to address the potential risks associated with repeated CT and PET/CT scans over several years, including radiation exposure [8] and the use of contrast agents [9]. While PET/CT is superior to cross-sectional imaging for melanoma staging [10], it exposes patients to higher radiation doses compared to CT [11]. Whole-body magnetic resonance imaging (WB-MRI) holds promise as a noninvasive diagnostic method without the risk of radiation exposure. However, neither the American [12] nor the European [5] guidelines for melanoma diagnostics currently mention WB-MRI as an alternative for whole-body imaging. Nevertheless, studies evaluating WB-MRI for cancer staging [13,14,15], specifically focusing on melanoma staging [10,16,17,18,19,20,21,22], have shown promising results. Additionally, advancements in MRI techniques, such as diffusion-weighted imaging (DWI), have contributed to an improved diagnostic sensitivity and specificity for malignant lesions [15,16,21]. Therefore, a reevaluation of the diagnostic performance of WB-MRI in comparison to CT and PET/CT is necessary to determine its potential as an alternative imaging technique with sufficient accuracy for reliable melanoma staging while reducing the patients’ radiation exposure.

The aim of this study was to directly compare the diagnostic performance of WB-MRI and WB-CT for staging patients with unresectable metastasized melanoma. The highest possible reference standard was established by including ^18^F-FDG-PET/CT and ^18^F-FDG-PET/MRI performed on the same day along with follow-up examinations.

## 2. Methods

This is a retrospective evaluation of a prospectively conducted study, which was approved by the local ethics committee (code: 251/2012B01) and was registered at the German Clinical Trials Register (DRKS00013925). Informed consent was obtained from all patients for the utilization of their data in research. The prospective study included patients who had clinically diagnosed unresectable metastasized melanoma and who were scheduled for systemic treatment. Exclusion criteria encompassed contraindications for MR-imaging (such as metal implants) and the use of gadolinium-based contrast agents. Additionally, patients with other acute illnesses, pregnant or breastfeeding women, and individuals unable to provide informed consent were excluded [23,24].

### 2.1. Patient Cohort

The initial study enrolled 62 patients with advanced (stage IV) unresectable melanoma. All participants underwent contrast-enhanced WB-^18^F-FDG-PET/CT and subsequently underwent WB-^18^F-FDG-PET/MRI on the same day between April 2014 and April 2018. In total, 3 out of the 62 patients were excluded from the study due to a lack of tracer uptake in the ^18^F-FDG-PET scan. Additionally, two other patients were excluded due to incomplete MRI scans. Patient characteristics of the remaining 57 patients are presented in Table 1.

### 2.2. PET/CT Examinations

All PET/CT examinations were performed on a state-of-the art clinical scanner (Biograph mCT^®^, Siemens Healthineers, Erlangen, Germany). All patients fasted for at least 6 h before examination. Weight-adapted (300–350 MBq) ^18^F-FDG was injected intravenously 60 min prior to image acquisition. Standardized CT examination protocols included weight-adapted (90–120 mL) intravenous CT contrast agent (Ultravist 370^®^, Bayer Vital GmbH, Leverkusen, Germany). Portal-venous phase acquisitions were obtained in expiration with 70 s delay time using a tube voltage of 120 kV and a reference dose of 200 mAs. Additional lung scans were performed during inspiration. PET was acquired from the skull base to the midthigh level over six to eight bed positions and was reconstructed using a 3D ordered subset expectation maximization algorithm (2 iterations, 21 subsets, Gaussian filter of 2.0 mm, matrix size of 400 × 400, and slice thickness of 2.0 mm). The PET acquisition time was two minutes per bed position.

### 2.3. PET/MRI Examinations

All patients were examined in a fully integrated 3 Tesla PET/MRI system (Biograph mMR, Siemens Healthineers GmbH, Erlangen, Germany). Routine PET/CT scans were performed prior to the PET/MRI examinations, leading to ^18^F-FDG uptake times for PET/MRI of about 120 min. All patients received a whole-body PET scan (head to thighs), which was acquired with scan emission times of 4 min per bed position. PET images were reconstructed using the vendor’s software with three-dimensional (3D) ordered subset expectation maximization algorithm, 21 subsets, 2 iterations, 256 × 256 matrix size (voxel size of 2.8 × 2.8 × 2.0 mm^3^), and 4 mm Gaussian filter. A 3D T1-weighted spoiled gradient-echo sequence with dixon-based fat–water separation in end-expiratory breath hold was acquired to create an attenuation map. All attenuation maps were checked carefully for erroneous tissue identification. We acquired HASTE (Half-Fourier Acquisition Single-Shot Turbo Spin Echo) imaging sequences during free breathing. A fat-saturated postcontrast T1 volume-interpolated breath-hold examination (VIBE) was acquired in axial view with multiple breath holds and the following parameters: voxel size of 1.7 × 1.7 × 3 mm^3^, slice thickness of 3 mm, and TR/TE of 3.97/1.26 ms. Gadobutrol (1.0 mmol/mL of Gadovist, Bayer Vital, Leverkusen, Germany) was used as contrast agent. The DWI sequence was acquired with the following parameters: image matrix of 192 × 168, slice thickness of 5 mm, no. of averages of 3, and b-values of 50 and 800 s/mm^2^. The apparent diffusion coefficient (ADC) maps were calculated by the vendor software.

### 2.4. Data Analysis

Two radiologists (3 and 6 years of experience) independently evaluated the CT and MRI examinations in a blinded manner without access to the results of the PET scans or previous/follow-up examinations. The MRI evaluations included the axial T1-VIBE, DWI, and HASTE sequences. In half of the patients, the CT readings were conducted before the MRI readings, while, in the other half, the MRI readings were performed first. To ensure the independence of the readings, there was a two-month time gap between the evaluation of the CT and MRI scans for each patient, preventing any influence from the results of the initial reading on the second reading. The assessment of malignant lesions was based on standard reading criteria, considering morphologic features and enhancement characteristics after application of contrast agents. Lymph nodes were considered metastatic if the short-axis measurement exceeded 10 mm, following the guidelines of RECIST 1.1 [25].

Lesions were categorized based on the following anatomical regions:

- Lymph nodes and soft tissue (e.g., muscle)
- Lungs (including pleural lesions)
- Abdomen/pelvis (including lesions in abdominal organs and peritoneal lesions)
- Bone

In the case of less than 10 lesions per organ, all findings were individually documented on a lesion-based basis. However, if there were more than 10 lesions per organ, they were documented with a cluster-based approach. For the site-based analysis, we combined the results from both readers, which closely resembled routine diagnostic procedures.

### 2.5. Standard of Reference

To establish a standard of reference, all PET/CT and PET/MRI examinations were assessed by a nuclear medicine physician and an experienced senior radiologist specializing in hybrid imaging. They reached a consensus regarding the evaluation of all lesions by considering previous or follow-up examinations, relevant clinical information, and histological results. The documentation of lesions followed the same anatomical classification system used for the CT and MRI readings.

### 2.6. Statistical Analysis

The comparative analysis between CT and MRI involved examining all documented findings using both a lesion-based method and a region-based method. Additionally, a third method, called the site-based method, was employed. In this method, the presence or absence of lesions per region was encoded as “0” or “1”, respectively. The number of true-positive, true-negative, false-positive, and false-negative findings were determined, allowing for the calculation of the sensitivity and specificity of each method. The comparative analysis was conducted separately for each reader. The inter-reader agreement was assessed using Bland–Altman procedures for each method and anatomical region. Furthermore, a cumulative overall reader was created to evaluate the overall diagnostic performance of CT and MRI. This involved integrating the results from both readers, with a negative finding recorded only if both readers did not identify a lesion in a region. Conversely, if at least one reader documented a lesion, a positive finding was noted. The use of a cumulative overall reader better reflects the typical clinical practice in hospitals, where CT and MRI examinations are usually evaluated by two radiologists. Statistical significance was determined by calculating *p*-values using McNemar’s test for dependent variables, with values less than 0.05 considered statistically significant. The statistical analyses and graphical representations were performed using the R software (version 4.1.2).

## 3. Results

### 3.1. Distribution of Metastases

Metastases were observed and documented in accordance with the reference standard for all the patients. Among the 57 patients, 4 had metastases exclusively in the lymph nodes and soft tissue, while 3 patients had metastases solely in the lungs. A total of 50 patients exhibited metastases in at least two anatomical regions. Table 2 provides an overview of the overall number of metastatic lesions as determined by the reference standard along with their distribution across different anatomical regions and the corresponding number of affected patients. Notably, the lymph nodes and soft tissue exhibited the highest occurrence of both individual and clustered malignant lesions.

### 3.2. Comparative Analysis

**Figure 1:**
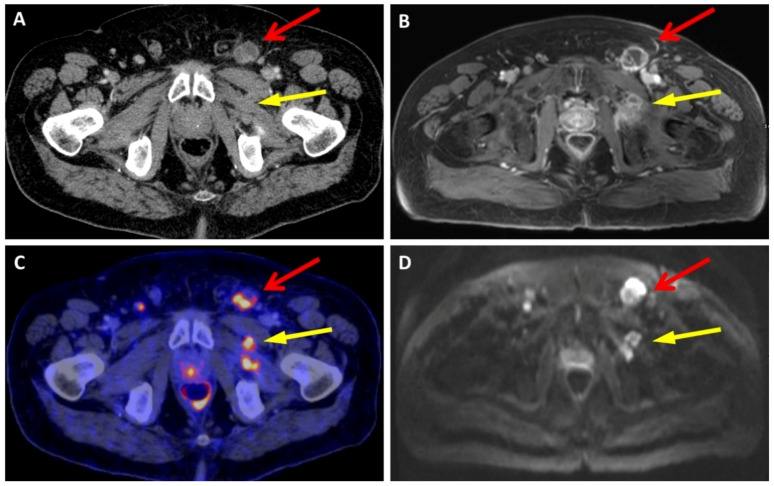
This patient exhibited metastatic lymph nodes in the left inguinal region (indicated by the red arrow) and metastatic soft-tissue lesions in the left M. obturatorius externus (highlighted by the yellow arrow), which could be distinguished using all imaging modalities. (**A**) is CT, (**B**) is VIBE, (**C**) is PET/CT, and (**D**) is HASTE.

In the “lymph nodes and soft tissue” region, there were no significant differences in diagnostic accuracy between CT and MRI, with both demonstrating a comparable sensitivity (0.88). However, there was a slight tendency towards a lower specificity in MRI (0.76 vs. 1.0) (Figure 1).

**Figure 2:**
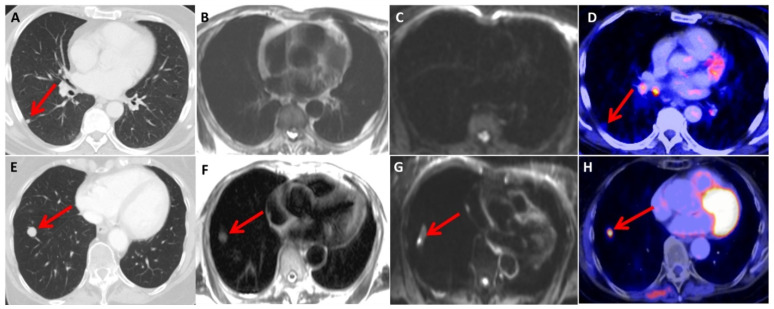
Two melanoma patients (upper row and lower row) presenting with pulmonary metastatic lesions (indicated by the red arrow) in the right lower lobe of the lungs. In the first patient (upper row), the metastasis is visible in the CT scan (**A**) but not in the HASTE (**B**) and DWI (**C**) images. A moderate increase in [18F]FDG uptake is observed in the PET scan (**D**). The second patient (lower row) had a comparatively larger metastasis, which is visible in the CT scan (**E**), HASTE (**F**) image, and DWI (**G**) image and exhibits intense [18F]FDG accumulation (**H**).

CT exhibited a significantly higher sensitivity for detecting lung lesions (*p* = 0.008) compared to MRI, with values of 0.90 and 0.68, respectively (Figure 2).

**Figure 3:**
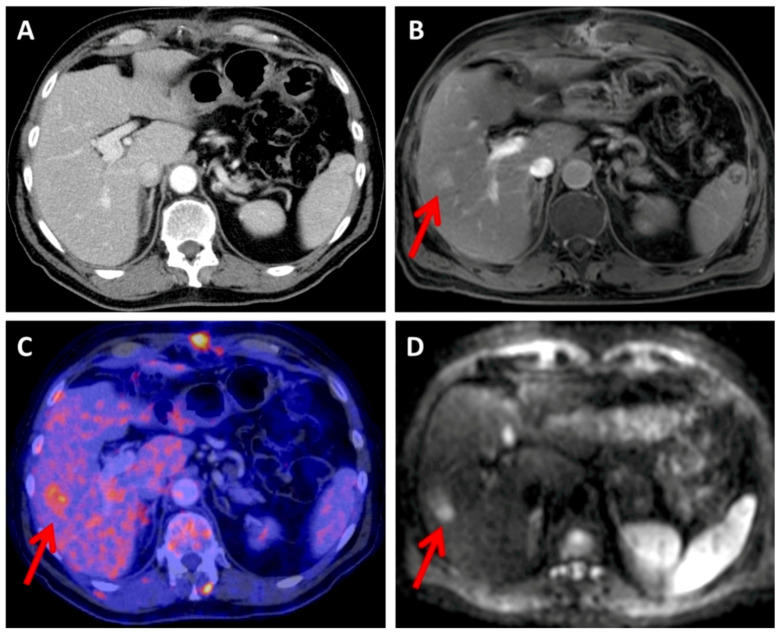
The melanoma patient had a metastatic hepatic lesion located in segment VIII (indicated by the red arrow), which is distinguishable in contrast-enhanced T1 VIBE (**B**) and DWI (**D**) images but is not visible in the CT scan (**A**). The lesion exhibits an elevated FDG uptake in the PET scan (**C**).

The detectability of organ metastases in the abdomen and pelvis, as well as bone metastases, was similar between CT and MRI (Figure 3).

**Figure 4:**
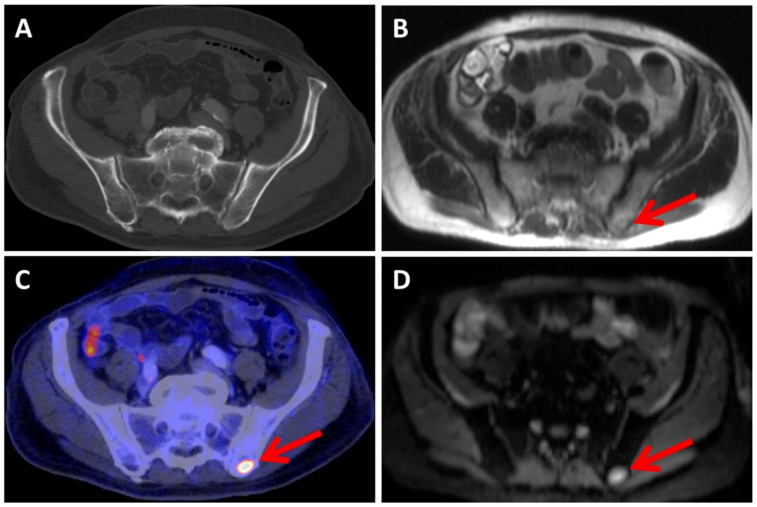
The patient presented with a metastatic bone lesion located in the left ilium (indicated by the red arrow), which is distinguishable in MRI using the HASTE sequence (**B**), DWI (**D**), and PET (**C**) imaging but is not visible in the CT scan (**A**).

The majority of false-negative findings were observed in the MRI readings of lung lesions (*n* = 10) and in the CT readings of bone lesions (*n* = 7) (Figure 4).

False-positive findings were most commonly found in the CT readings of lymph-node and soft-tissue lesions (*n* = 7) and in the MRI readings of bone lesions (*n* = 5). Table 3 provides an overview of the number of positive findings, regardless of the number of metastases, as determined by the reference standard and compares them to the results obtained by the combined reader for CT and MRI.

### 3.3. Inter-Reader Reliability

The level of agreement between the readers varied depending on the number of metastases and the anatomical region. When evaluating CT readings, there was a noticeable absence of statistically significant proportional bias and significant bias only in the cases of single metastases in the lung, single metastases in the abdomen/pelvis, and metastatic clusters (consisting of more than 10 lesions) in the skeleton. Among the different anatomical regions, the readings for the abdomen/pelvis region exhibited the lowest interrater variability, while the readings for the lymph nodes/soft tissue had the highest inter-reader reliability, indicating the lowest agreement between the readers. Regarding specific anatomical regions, a high level of agreement between the readers was achieved in the detection of more than 10 lesions in the lung with CT (Fleiss’ kappa = 0.82) and in the detection of more than 10 bone lesions with MRI (Fleiss’ kappa = 0.81). However, there was a significant proportional bias in the MRI readings for lung and abdominal/pelvic lesions as well as in the CT readings for bone lesions. Table 4 provides a summary of the results regarding inter-reader reliability.

## 4. Discussion

In this study, we directly compared the diagnostic performance of WB-MRI and WB-CT performed on the same day for staging melanoma patients. The comparison focused on lesion detection rates and inter-reader reliability, using a reference standard that included PET.

There was no significant difference between CT and MRI in detecting metastases in the lymph nodes, soft tissue, and muscle when compared to the reference standard. However, MRI exhibited a lower detection rate for pulmonary metastases (sensitivity of 0.68, specificity of 0.93) compared to CT (sensitivity of 0.90, specificity of 0.86). On the other hand, MRI showed a higher detection rate for bone lesions (sensitivity of 0.89, specificity of 0.93) compared to CT (sensitivity of 0.61, specificity of 0.88), but this difference was not statistically significant. The variability in the number of metastases detected through CT and MRI, relative to the reference standard, depended on the anatomical region and the number of lesions per patient.

CT is the established staging method for monitoring oncology patients, including those with malignant melanoma, due to its short acquisition time and widespread availability. However, repeated CT examinations may increase the risk of developing secondary malignancies due to radiation exposure, which is particularly relevant for young melanoma patients undergoing regular follow-ups [8]. For a 50-year-old patient, it is estimated that annual CT scans of the chest, abdomen, and pelvis over a 10-year period carry a lifetime cancer risk of 0.9% to 1.3% [26]. Improved long-term survival rates have been achieved through new targeted therapies, such as mitogen-activated protein kinase (MAPK) pathway inhibitors and inhibitors of cytotoxic-T-lymphocyte-associated protein 4 (CTLA4) and programmed cell death 1 (PD1)/PD1L [27,28,29,30]. Given the increasing importance of reducing radiation exposure, it is necessary to reevaluate the imaging strategies for staging and follow-up with respect to melanoma patients [6,31].

Another concern relates to the use of iodinated intravenous CT contrast agents, which can pose risks such as hyperthyroidism, potential allergic reactions, and renal toxicity, especially in patients with pre-existing renal function impairment or congestive heart failure [9]. Significant advancements in MRI technology, including improvements in hardware (such as a higher field strength, more powerful gradients, and advanced matrix coil systems), software, and examination sequences (focused on reducing artifacts, acceleration techniques, compressed sensing, and parallel acquisition technology), as well as developments in quantitative imaging (such as diffusion-weighted imaging, MR fingerprinting, and mapping) have continuously enhanced the clinical applicability of this radiation-free imaging modality [32]. WB-MRI scans can now be performed with high resolution and reasonable examination times, which is particularly advantageous for diffusion-weighted imaging. This technique allows for a more accurate and specific characterization of the tissue microstructure, thereby increasing its clinical value in oncological imaging [18]. As a result of these advancements, WB-MRI has emerged as a competitive alternative to WB-CT for the staging of patients with advanced melanoma. This is particularly significant considering the potential risks associated with CT contrast agents and the increasing clinical utility of MRI in providing detailed and radiation-free imaging.

Other studies have also reported that MRI is less sensitive than CT in detecting pulmonary metastases [15,18]. In particular, the upper and lower regions of the lungs may be affected by respiratory artifacts, leading to an increased risk of false-negative results when pulmonary lesions smaller than 10 mm are not identified. The technical complexity of WB-MRI and the absence of standardized examination protocols across different institutions are additional limiting factors that have not yet been validated. However, the development of MRI ultrashort echo time (UTE) techniques has shown promise in improving the assessment of small pulmonary nodules. These techniques allow for an echo time (TE) shorter than 200 μs, resulting in a significantly higher sensitivity compared to conventional volume-interpolated breath-hold examination (VIBE) images [33].

Patients who have limited pulmonary involvement with isolated or focal masses may be candidates for a surgical metastasectomy. Studies have shown that patients who undergo complete resections of their pulmonary metastases have improved 5- and 10-year survival rates of 22% and 16%, respectively [34].

In contrast to previous studies [21,22], our study did not find a higher sensitivity with the detection of abdominal metastases using MRI. This discrepancy may be explained by our site-based analysis, where we did not differentiate between individual abdominal organs, such as the liver, and peritoneal lesions. While MRI exhibits an excellent sensitivity for detecting liver lesions, its sensitivity for peritoneal lesions is only moderate. On the other hand, MRI demonstrated a higher sensitivity and specificity for the detection of bone lesions, although this difference was not statistically significant in our cohort. The superiority of MRI in assessing bone lesions has also been reported by other authors [19,20,35].

The diagnostic accuracies of MRI and CT for detecting metastatic lesions in the lymph nodes, soft tissue, and muscle were found to be similar. When it comes to the lymph nodes, CT is generally considered more sensitive. However, MRI is known for its superior soft-tissue contrast, resulting in a higher sensitivity for subcutaneous and muscle lesions [18]. It is worth noting that, in this site-based analysis, the grouping of lymph-node, soft-tissue, and muscle lesions into a single region may have influenced our findings.

In our study, we observed a higher agreement between the readers for metastatic clusters (comprising more than 10 metastases per region) in the LSM (both CT and MRI), lungs (CT), A/P (CT), and bone (MRI). Overall, the inter-reader reliability was highest for metastases in the A/P region, while it was lowest for the lymph nodes, soft tissue, and muscle. These results align with the site-based sensitivity of each imaging method. In MRI scans, detecting lung lesions and peritoneal lesions proved more challenging, leading to discrepancies in detection rates between the readers. The same holds true for bone lesions in CT scans, as they are only visible in cases of sclerosis or osteolysis.

Given that WB-MRI demonstrates a comparable accuracy to other methods for melanoma staging, except for lung evaluation, it could be beneficial to combine WB-MRI with low-dose chest CT. Low-dose chest CT without a contrast agent has shown equivalent diagnostic performance in detecting lung nodules when compared to standard chest CT [36]. Incorporating artificial intelligence (AI) applications into the clinical practice has the potential to enhance advanced quantitative imaging techniques such as magnetic resonance fingerprinting and radiomics [32]. In the context of personalized medicine, with the increasing economic pressures and workload, it becomes imperative to standardize oncological MR imaging to ensure the consistency and reliability of the results [37].

Our study has limitations. First, we did not include an evaluation of the central nervous system in our analysis. MRI is known for its ability to detect small asymptomatic brain metastases, which have a significant impact on the prognosis of stage IV melanoma patients [38]. However, since our institution typically performs dedicated brain MRI due to the acknowledged superiority of MRI over CT in detecting brain and spinal-cord lesions [39], it would be advantageous to incorporate brain examinations into a WB-MRI approach. Second, we acknowledge that a double read approach may lead to an overestimation of the performance of CT and MRI. However, it is important to note that, in our study, we conducted a comparative analysis of CT and MRI, employing the double read approach equally for both imaging techniques. Third, not all of the lesions identified in PET/CT and PET/MRI could undergo histological verification. To address this, we included additional follow-up examinations and considered the long-term clinical outcomes to determine whether the lesions should be classified as benign or malignant. Fourth, it has been demonstrated that assessing a large number of lesions per region increases the risk of discrepancies between different raters. Nevertheless, this is a common procedure in everyday clinical practice.

In summary, our study findings support the potential of WB-MRI as a viable alternative to CT for staging melanoma patients, offering comparable diagnostic accuracies and confidences in most regions. This has the advantage of significantly reducing radiation exposure, which is particularly valuable given the improved clinical outcomes associated with immunotherapy. To address the observed limited sensitivity for detecting pulmonary lesions, the implementation of specific sequences in lung imaging could potentially enhance its performance.

## Figures and Tables

**Table 1:** Patient characteristics.

Patient Characteristics	*n*
Sex	
Male	32
Female	25
Age	
Mean	63
Range	49–84
Histologic type	
SSM	13
NM	9
ALM	6
UM	5
MM	9
OM	6
Others	9
Clinical stage	
IV	57 (100%)

Abbreviations: SSN = superficial spreading melanoma; NM = nodular melanoma; ALM = acral lentiginous melanoma; UM = uveal melanoma; MM = malignant melanoma (no type defined); OM = occult melanoma; others include nevus-associated melanoma, nevoid melanoma, mucosal melanoma, and spitzoid melanoma.

**Table 2:** The total counts of metastatic lesions and metastatic clusters (consisting of more than 10 lesions per region) per anatomical region were categorized based on the reference standard (Ref.), CT, and MRI. For each method, a hypothetical cumulative reader was employed for evaluation.

	Metastases (*n* < 10)			Metastatic Clusters (*n* > 10)			No. of Patients
	Ref.	CT	MRI	Ref.	CT	MRI	
Lymph nodes, soft tissue	247	91.2	77.1	22	28.8	27.0	41
Lungs	57	36.3	21.4	16	10.4	9.8	27
Abdomen, Pelvis	82	61.6	47.0	20	14.7	14.0	29
Bone	25	13.8	29.9	9	5.9	5.9	38

**Table 3:** In a site-based analysis, the number of true-positive (TP), false-positive (FP), and false-negative (FN) findings along with the positive predictive value (PPV), negative predictive value (NPV), sensitivity, and specificity of CT and MRI were compared to the reference standard (Ref.).

	No. of Findings (Ref.)	Modality	TP	FP	FN	PPV	NPV	Sens.	Spec.	*p* Value (CT vs. MRI)
Lymph nodes/soft tissue/muscle	42	CT	37	0	5	1.00	0.77	0.88	1.00	0.221
		MRI	37	4	5	0.90	0.72	0.88	0.76	
Lungs	31	CT	28	4	3	0.88	0.89	0.90	0.86	0.008
		MRI	21	2	10	0.91	0.72	0.68	0.93	
Abdomen/pelvis	35	CT	31	3	4	0.91	0.84	0.89	0.88	0.343
		MRI	27	3	8	0.90	0.72	0.77	0.88	
Bone	18	CT	11	5	7	0.69	0.84	0.61	0.88	0.606
		MRI	16	3	2	0.84	0.95	0.89	0.93	

**Table 4:** The inter-reader reliability was assessed for the number of lesions, categorized as <10 and >10 detected lesions, when comparing CT and MR scans across various regions. Proportional bias (PB), Fleiss’ kappa (FK), Pearson correlation (PK), and Spearman’s ρ were used as measures in the analysis.

	Lymph Nodes/ Soft Tissue/Muscle				Lung				Abdomen/Pelvis				Bone			
	CT		MR		CT		MR		CT		MR		CT		MR	
No. of lesions	<10	>10	<10	>10	<10	>10	<10	>10	<10	>10	<10	>10	<10	>10	<10	>10
Proportional bias	1.27	0.76	1.26	0.95	1.37	0.80	1.54	1.76	1.21	0.86	1.44	1.44	0.61	1.23	1.12	0.85
Fleiss’ Kappa	0.21	0.46	0.30	0.64	0.44	0.82	0.51	0.54	0.48	0.59	0.39	0.39	0.38	0.44	0.38	0.81
Pearson Correlation	0.44	0.62	0.36	0.83	0.38	0.87	0.25	0.50	0.50	0.80	0.33	0.33	0.82	0.45	0.64	0.83
Spearman’s ρ	0.50	0.62	0.40	0.83	0.63	0.89	0.62	0.62	0.68	0.70	0.66	0.66	0.70	0.45	0.66	0.83

## Data Availability

The data presented in this study are available on request from the corresponding author. The data are not publicly available due to ethical restrictions.

## Abbreviations

DWI	Diffusion-weighed imaging
^18^F-FDG	^18^F-fluorodeoxyglucose
HASTE	Half-Fourier Acquisition Single-Shot Turbo Spin Echo
MAPK	Mitogen-activated protein kinase pathway
CTLA4	Cytotoxic-T-lymphocyte-associated protein 4
MBq	Megabecquerel
PD1	Programmed cell death 1
PET	Positron emission tomography
UTE	Ultrashort echo time
RECIST	Response Evaluation Criteria In Solid Tumors
VIBE	Volume-interpolated breath-hold examination
WB	Whole-body
